# Early dynamic left intraventricular obstruction is associated with hypovolemia and high mortality in septic shock patients

**DOI:** 10.1186/s13054-015-0980-z

**Published:** 2015-06-17

**Authors:** Jean-Louis Chauvet, Shari El-Dash, Olivier Delastre, Bernard Bouffandeau, Dominique Jusserand, Jean-Baptiste Michot, Fabrice Bauer, Julien Maizel, Michel Slama

**Affiliations:** General Intensive Care Unit, Elbeuf Intercommunal Hospital Center, Elbeuf, France; Service de Réanimation Médicale, CHU Sud 80054, cedex 1, France; LIM-09 Medical Research Laboratory in Experimental Pneumology, Faculty of Medicine of the University of São Paulo, São Paulo, Brazil; Heart Failure and pulmonary hypertension Clinic, Echo Core Lab, Cardiology Department, Charles Nicolle Hospital, Rouen University Hospital, Rouen, France; INSERM U-1088, Jules Verne University of Picardie, Amiens, France

## Abstract

**Introduction:**

Based on previously published case reports demonstrating dynamic left intraventricular obstruction (IVO) triggered by hypovolemia or catecholamines, this study aimed to establish: (1) IVO occurrence in septic shock patients; (2) correlation between the intraventricular gradient and volume status and fluid responsiveness; and (3) mortality rate.

**Method:**

We prospectively analyzed patients with septic shock admitted to a general ICU over a 28-month period who presented Doppler signs of IVO. Clinical characteristics and hemodynamic parameters as well as echocardiographic data regarding left ventricular function, size, and calculated mass, and left ventricular outflow Doppler pattern and velocity before and after fluid infusions were recorded.

**Results:**

During the study period, 218 patients with septic shock were admitted to our ICU. IVO was observed in 47 (22 %) patients. Mortality rate at 28 days was found to be higher in patients with than in patients without IVO (55 % versus 33 %, *p* < 0.01). Small, hypercontractile left ventricles (end-diastolic left ventricular surface 4.7 ± 2.1 cm^2^/m^2^ and ejection fraction 82 ± 12 %), and frequent pseudohypertrophy were found in these patients. A rise ≥12 % in stroke index was found in 87 % of patients with IVO, with a drop of 47 % in IVO after fluid infusion.

**Conclusion:**

Left IVO is a frequent event in septic shock patients with an important correlation with fluid responsiveness. The mortality rate was found to be higher in these patients in comparison with patients without obstruction.

## Introduction

Left intraventricular flow obstruction (IVO) is classically described in asymmetric hypertrophic cardiomyopathy [1, 2] and is characterized by a saber-shaped Doppler flow curve, with late acceleration [3]. The obstruction is usually at the level of the left ventricular outflow tract (LVOT), and is due to systolic anterior movement (SAM) of the anterior leaflet of the mitral valve. This phenomenon has also been previously described in certain clinical situations outside the setting of hypertrophic cardiomyopathy, mainly revolving around hypovolemia and catecholamine exposure [4–12]. On the other hand, the early phase of septic shock is associated with hypovolemia, hyperkinesia and low left ventricular (LV) afterload (making catecholamine infusion necessary), which are hemodynamical situations which may induce IVO. Nevertheless, there has been no previous study of the incidence of left ventricular obstruction in septic shock, or of the fluid responsiveness of patients presenting with this obstructive flow pattern. Furthermore, clinical consequences of this obstruction have not been analyzed.

The objective of this prospective study was thus to gather information prospectively about the prevalence and clinical implications of this kind of flow pattern (IVO) in the septic shock patients admitted to our ICU.

## Methods

### Patients

It is standard practice in our unit to perform an echocardiographic assessment of hemodynamically unstable patients. All examinations are performed by one of three trained staff members and imaging is repeated at all therapy changes, and after every 500 ml of fluid loading in patients requiring intravascular expansion. Due to a prior interest in the phenomena, during the 28-month period from April 2008 to October 2010, whenever a patient’s echocardiogram suggested an IVO obstruction (peak velocity LVOT ≥0.9 m/s), we prospectively stored all their two-dimensional images and Doppler curves in a designated patient file for later review or retrieval, and documented the clinical information documented on the medical records and ICU observation charts for the same date and time. All echocardiographic examinations were performed during the first 6 hours following admission to the ICU and all septic shock patients had an echocardiographic study. This information included demographic data (such as age, sex, diagnosis, Simplified Acute Physiology Score (SAPS) II, presence of mechanical ventilation, ICU stay, ICU mortality, and mortality at 28 days), as well as physiological variables (such as heart rate, systolic blood pressure, diastolic blood pressure, mean arterial pressure, pulse pressure, pulse pressure variations, catecholamine use and dose, hemodialysis), and ventilator parameters (such as presence of mechanical ventilation and tidal volume).

We analyzed baseline characteristics and mortality of all patients with septic shock without IVO hospitalized during the same period. All patients in this group had an early echocardiography examination that allowed us to rule out IVO; however, images were not stored for patients without obstruction and therefore were unavailable for complete analysis in this study.

This study was approved by the ethics committee of Rouen hospital, and the research committee waived consent due to the observational nature of the study.

### Echocardiography data

From the patients’ echocardiography examinations we collected information regarding left ventricular (LV) size and function. From the parasternal long axis view using M-mode we measured the left ventricular end-diastolic diameter (LVEDD), and septal (S) and posterior (P) wall thickness. From the parasternal short axis view we measured the LV area in diastole and in systole and then we calculated the shortening fractional area. LV mass was calculated using the formula 0.8 × (1.04 × (LVEDD + S + P)^3^ – LVEDD^3^) – 13.6 g. LV mass was considered normal if <115 g/m^2^ in males and <115 g/m^2^ in females. Pseudohypertrophy was defined as LV wall thickness ≥12 mm with a normal calculated LV mass. Inferior vena cava diameter was obtained from a subcostal view, mitral flow velocities (E and A wave) using pulsed Doppler from an apical four-chambers view, annulus velocities (E’ and A’) from an apical four-chambers view using tissue Doppler imaging, as well as LV outflow Doppler data (velocity and curve morphology) in patients with IVO using continuous wave Doppler. The location of IVO was analyzed and found at the level of the LVOT or at the level of the midventricle. We considered a mitral regurgitation as moderate or severe if regurgitant flow width was >6 mm. All these parameters were recorded during the end-expiratory phase. Cardiac output was calculated from measurements of aortic annulus measured from the parasternal long axis view and aortic VTI measured on aortic blood flow recorded using pulsed Doppler at the level of the aortic annulus from an apical four-chambers view. Information from each examination performed before a fluid bolus was compared with data from the corresponding “after-bolus” examination.

### Statistics

All data are presented as mean ± SD. Student's t test was performed to compare continuous values and chi-squared test performed for percentages. Analysis of variance for repeated measurement was used to compare pre- and postfluid measurements. Statistical significance was defined as a *p*-value less than 0.05. A Kaplan-Meier survival curve was done for the mortality rate. Multivariable logistic regression was also performed to analyze the effect of IVO. The results are shown as odds ratios with 95 % confidence intervals. Two-sided p-values less than 0.05 were considered statistically significant.

## Results

**Fig. 1:**
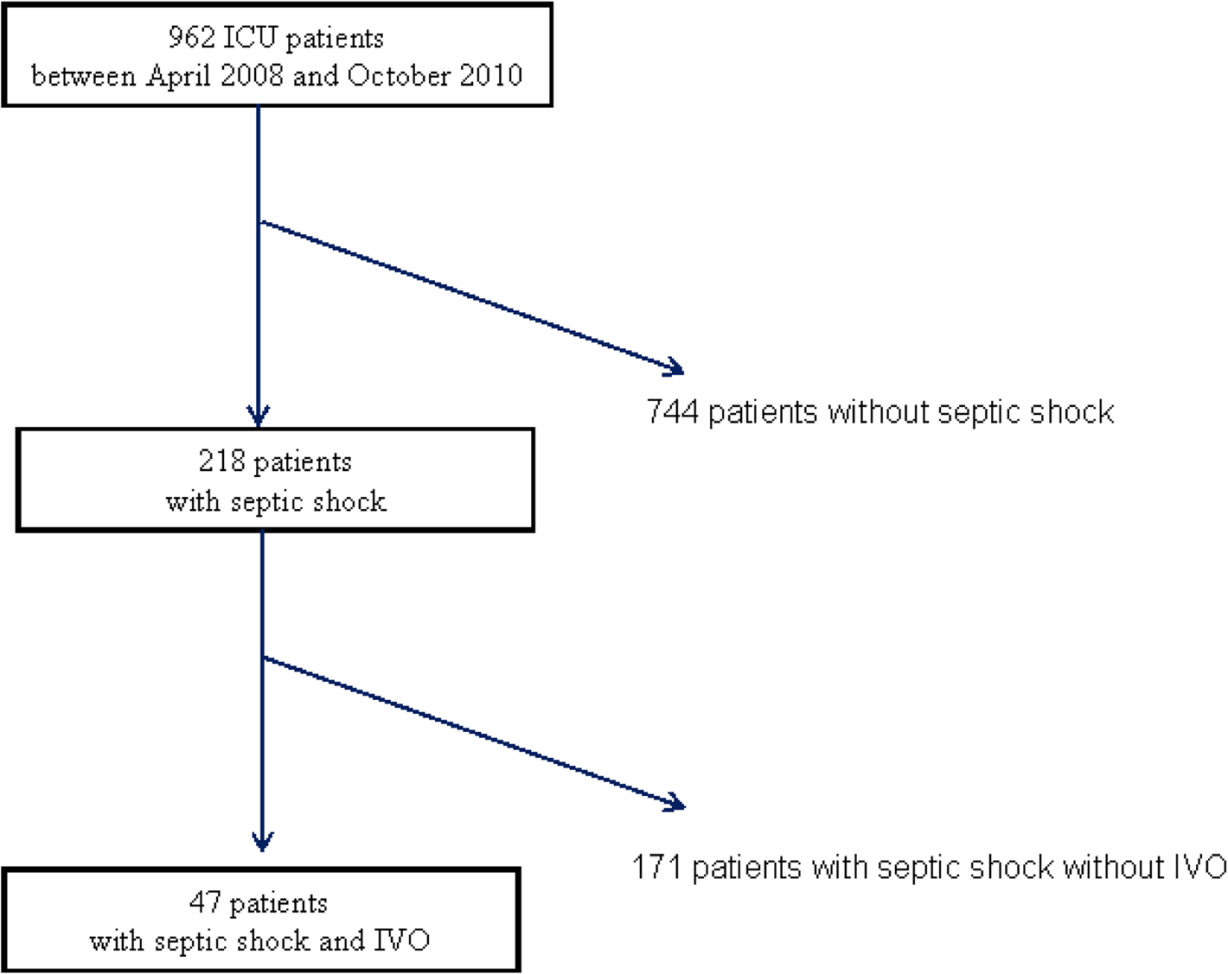
Flow chart. *IVO* intraventricular obstruction

During the 28 month timeframe, 218 septic shock patients were admitted to the ICU, of which a total of 47 (22 %) had a documented IVO flow pattern (Fig. 1).

### Baseline characteristics

A comparison of the baseline characteristics between the patients with or without LVO is presented in Table 1. Age, SAPS II, length of stay, the proportion of males, mechanical ventilation, and the numbers of medical and surgical patients were similar between both groups. Thirty percent of patients had a diagnosis of acute respiratory distress syndrome in both groups with and without IVO.

**Table 1:** Characteristics of patients with septic shock with and without IVO

	Septic shock patients without IVO (n = 171)	Septic shock patients with IVO (n = 47)	*p* value
Age (years)	62 ± 15	69 ± 11	ns
Males	106 (62 %)	27 (57 %)	ns
SAPS II	54 ± 21	59 ± 16	ns
Mechanical ventilation	149 (87 %)	39 (83 %)	ns
ICU stay duration (days)	11 ± 27	12 ± 10	ns
Medical patients	109 (64 %)	32 (68 %)	ns
Surgical patients	62 (36 %)	15 (32 %)	ns
ICU mortality	41 (24 %)	25 (53 %)	<0.01
Mortality at 28 days	57 (33 %)	26 (55 %)	<0.01

Values are shown as n (%) or mean ± SD. *IVO* intraventricular obstruction, *ns* not significant, *SAPS* Simplified Acute Physiology Score

A total of 47 patients with septic shock with left ventricular obstruction were analyzed. Twenty-seven patients (57 %) were male. The mean age was 69 ± 11 years (range 37 to 85); the mean SAPS II score [13] was 59 ± 16 (range 30 to 97), and the mean body mass index was 29 ± 7 kg/m^2^ (range 16 to 52). Average length of stay in the ICU was 12 ± 10 days (range 1 to 51).

The cause of sepsis was divided between pneumonia (51 %), peritonitis (26 %), cellulitis (9 %), urinary infection (6 %), pericarditis (4 %), endocarditis (2 %) and pancreatitis (2 %), and was not statistically different in the group of septic shock patients without obstruction.

Forty three percent of these patients with IVO were on dialysis. In terms of ventilatory support, 17 % of them were spontaneously breathing, while 83 % were intubated, on volume-controlled ventilation or pressure support mode, with a mean tidal volume of 8 ± 1 ml/kg ideal body weight, and a mean PaO_2_/FiO_2_ ratio of 196 ± 101 mmHg. No difference was found compared to the group without IVO.

The mean lactate value obtained from records was 3.4 ± 3.5 mmol/l, and all patients were receiving at least one kind of catecholamine: 13 % of patients were receiving a dobutamine infusion at a mean dose of 8.8 ± 4.6 μg/kg/minute, while 83 % were receiving noradrenaline at 1.2 ± 1.6 μg/kg/minute, and 6 % of patients were on an adrenaline infusion at a rate of 1.1 ± 0.9 μg/kg/minute; there was no significant difference compared to the group without obstruction.

### Echocardiographic characteristics of patients with IVO

Transthoracic echocardiography examination was performed using Vivid-I (General Electric (GE), Fairfield, Connecticut, USA) early after the onset of the septic shock (2.5 ± 2.7 hours) within the first 6 hours in 31/47 (66 %) of patients, and the characteristics are shown in Table 2.

**Table 2:** Hemodynamic and echocardiographic characteristics of patients with intraventricular obstruction before and after fluid infusion

	Before	After	*p* value
Heart rate (beats/minute)	109 ± 25	102 ± 22	ns
Systolic arterial pressure (mmHg)	110 ± 28	141 ± 32	<0.01
Diastolic arterial pressure (mmHg)	54 ± 11	65 ± 14	<0.01
Mean arterial pressure (mmHg)	71 ± 16	89 ± 1	<0.01
Cardiac output (l/minute)	4.6 ± 1.9	5.8 ± 2.1	<0.01
Cardiac index (l/minute/m^2^)	2.4 ± 0.9	3 ± 1	<0.01
Stroke volume (ml)	43 ± 18	58 ± 22	<0.01
Indexed stroke volume (ml/m^2^)	23 ± 10	31 ± 11	<0.01
Intraventricular obstruction (m/s)	1.9 ± 0.9	1 ± 1	<0.01
Intraventricular obstruction (mmHg)	18 ± 18	8 ± 13	<0.01
Inferior vena cava diameter (mm)	16 ± 6.4	18 ± 6.6	ns
E/A	0.8 ± 0.46	0.9 ± 0.3	ns
E’ (cm/s)	14 ± 5	16 ± 5	ns
E’/A’	0.77 ± 0.26	0.87 ± 0.37	ns
E/E’	5.8 ± 2.1	6.8 ± 1.8	ns

*A* late velocity of diastolic mitral flow, *A’* late velocity of diastolic mitral annulus motion, *E* early diastolic velocity of mitral flow, *E’* early velocity of diastolic mitral annulus motion, *ns* not significant

#### Position of the intraventricular gradient

**Fig. 2:**
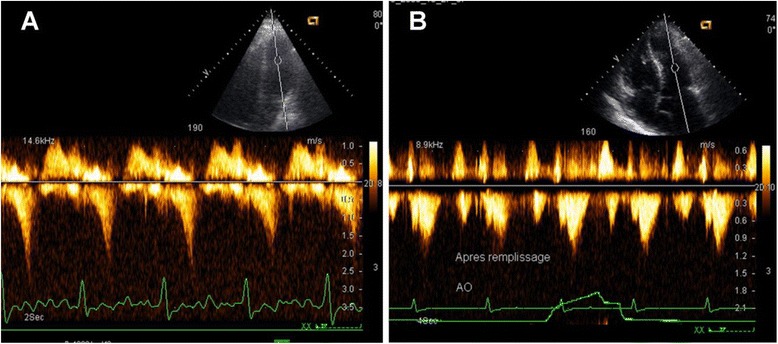
Left intraventricular obstruction. **a** Pulsed-wave Doppler curve showing the characteristic late peaking saber-shape indicating a LV outflow obstruction (IVG). **b** After fluid replacement, the flow profile returns to normal symmetrical shape

An intraventricular gradient (IVG) compatible with a mid-LV obstruction (Fig. 2) was noted in 17 patients, 11 patients had LV outflow obstruction, and 19 had both.

#### SAM of the mitral valve

Thirty one (66 %) patients had a SAM, and in 13 % of these cases (4/31) it was accompanied by significant mitral regurgitation (moderate or severe).

#### Evidence of hypovolemia

The patients with IVO were found to have small and hypercontractile left ventricles. End-diastolic LVEDD diameter was registered in 34 patients (72 %) and was found to be quite small, with a mean value of 37 ± 9 mm. Measurements of the indexed end-diastolic area of the left ventricle was also available in 22 patients (47 %) and had a mean value of 4.7 ± 2.1 cm^2^/m^2^. Hypercontractility with a LV ejection fraction (LVEF) ≥70 % was observed in 45 of the 47 patients (96 %). The mean LVEF across all 47 patients was 82 ± 12 %.

#### Small left ventricle and pseudohypertrophy

**Fig. 3:**
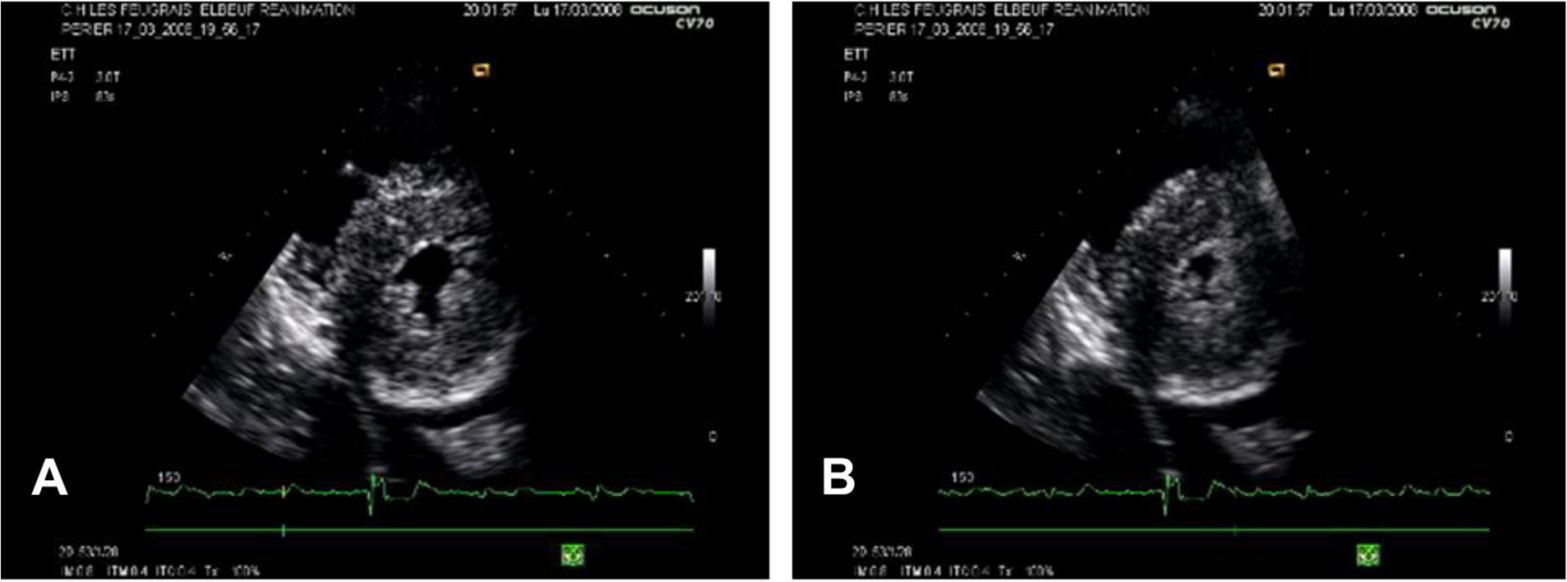
Pseudohypertrophy. Echocardiography in the parasternal short axis view. Ventricular wall appear thickened in these two-dimensional images, despite LV mass being unchanged. **a** The reduction in LVEDD size of the left ventricle (LV). **b** The near obliteration of the ventricular lumen in systole (“kissing” walls)

Though only two patients had previously diagnosed myocardial hypertrophy, 30 patients (64 %) exhibited thickened myocardial walls at the time of the first examination: 20 (43 %) had an end-diastolic posterior wall thickness ≥12 mm, 2 (4 %) had a septal end-diastolic thickness ≥13 mm and 9 (19 %) had a maximum thickness septal bulge ≥15 mm. In the majority of these patients (16 cases) we were able to calculate ventricular mass [14] and found that in 81 % of them (13 cases) the calculated LV mass was within the range of normality, despite the apparent thickening, and that, therefore, the wall thickening seen on two-dimensional echocardiography was actually pseudohypertrophy (Fig. 3).

### Pre- and postfluid bolus comparison

**Fig. 4:**
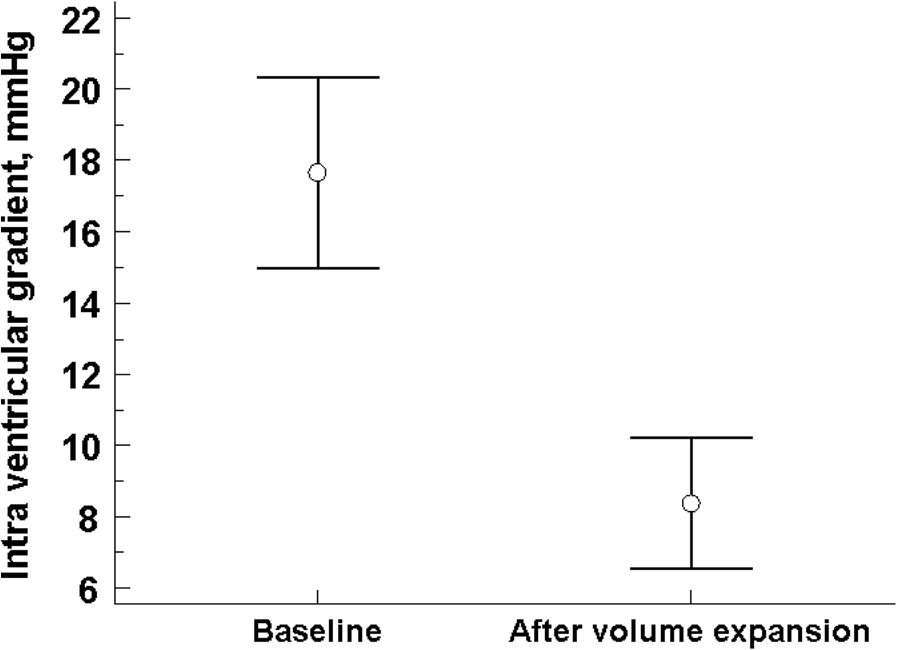
Pre- and postfluid bolus comparison. Comparison of intraventricular obstruction gradient before and after a single 500 ml fluid bolus; points correspond to mean values and error bars to two standard errors of the mean. The difference before and after volume expansion was statistically different (*p* = 0.001)

The mean IVG velocity prior to a first fluid bolus was 1.9 ± 0.9 m/s (Table 2). Postfluid bolus examination showed a disappearance of the IVG pattern in 17 patients (36 %). The mean IVG peak velocity after a single 500 ml fluid bolus was reduced to 1 ± 1 m/s, constituting an average drop of 0.9 m/s (or 47 %) (*p* < 0.01) (Fig. 4).

Cardiac index rose from 2.4 ± 0.9 to 3.0 ± 1.0 l/minute per m^2^ after fluid administration, thus increasing an average of 0.6 ± 0.5 l/minute per m^2^, or 31 ± 31 %, while the systolic index rose an average 8 ± 5 ml/m^2^, or 35 %, from 23 ± 9 to 31 ± 11 ml/m^2^ (*p* < 0.01).

Mean arterial pressure rose from 71 ± 16 to 89 ± 20 mmHg, an average increase of 18 ± 20 mmHg (30 %), and systolic blood pressure rose from 110 ± 28 to 141 ± 32 mmHg (*p* < 0.01). Heart rate dropped from 109 ± 25 to 102 ± 22 beats/minute, constituting a mean drop of 8 ± 14 beats/minute (5 %) after a single 500 ml fluid challenge (not significant).

### Fluid-responsiveness of patients with IVO

Thirty five of the 47 patients (79 %) had an increase ≥12 % in cardiac index; 40 patients (87 %) had an increase greater than 12 % in their stroke index (SI). Ninety eight percent of patients presented at least a 10 % rise in SI, and all patients increased their SI after volume infusion.

### Mortality

ICU mortality (Table 1) was statistically higher in the group of septic shock patients with LV obstruction when compared with patients without obstruction (25/47 (53 %) versus 41/171 (24 %), *p* < 0.01)) as was the mortality at 28 days (26/47 (55 %) versus 57/171 (33 %), *p* < 0.01). In multivariate analysis, compared to patients without IVO, the patients with IVO had an increased 28-day mortality (odds ratio 2.23, 95 % confidence interval 1.08–4.58, *p* = 0.03) and ICU mortality (odds ratio 3.77, 95 % confidence interval 1.77–8.03, *p* = 0.001) after adjusting for SAPS II (Table 3).

**Table 3:** Logistic regression analysis for 28-day mortality and ICU mortality

	28-day mortality			ICU mortality		
	OR	95 % CI	*p* value	OR	95 % CI	*p* value
SAPS II	1.06	1.04–1.08	0.001	1.06	1.04–1.09	0.001
Presence of IVO	2.23	1.08–4.58	0.03	3.77	1.77–8.03	0.001

*CI* confidence interval, *IVO* intraventricular obstruction, *OR* odds ratio, *SAPS* Simplified Acute Physiology Score

## Discussion

In this prospective study, IVO was seen in 22 % of patients with septic shock admitted to the ICU over the 18-month period studied.

The presence of IVO is associated with a high mortality rate. The data showed an association between the presence of this obstructive flow pattern on LV Doppler flow and the presence of hypovolemia and cardiac hypercontractility, and also demonstrated that these patients frequently present with small and pseudohypertrophic left ventricles. Furthermore, this study also demonstrates the high rate of fluid responsiveness in these patients with IVO despite the absence of significant pulse pressure variations.

Dynamic obstruction at the level of the LVOT was classically described in patients with congenital asymmetric left ventricular hypertrophy (LVH) [1–3], and is physiopathologically explained by a systolic narrowing of the LVOT creating a Venturi effect. This obstruction is usually associated with SAM of the anterior leaflet of the mitral valve into the LVOT [2, 4]. IVO is not limited to the classic septal hypertrophy and may also be seen in other congenital cardiomyopathies with mid-ventricular or apical hypertrophy [15, 16]. IVO has also been demonstrated in patients with concentric hypertrophy, such as that seen in poorly controlled arterial hypertension. Furthermore, some patients with LVH may have a latent (subclinical) obstruction which can be precipitated by hypovolemia, stress or by the administration of exogenous catecholamines such as adrenaline, noradrenaline or dobutamine, all of which may disturb this delicate balance [4–10]. Moreover, LVOT obstruction is also described in situations of impairment of apical contractility and relative basal hypercontractility, such as anterior wall infarcts or the apical ballooning syndrome, a condition in which high catecholamine levels are also implicated [8, 15, 17, 18]. Dynamic IVO may also appear in the postoperative setting, notably after aortic stenosis repair, or even after mitral valve repair, if the position of the mitral prosthesis anatomically interferes with the LVOT [19, 20]. Furthermore, several case reports illustrated that, even in the absence of LVH, obstruction can also develop in patients with acute intravascular fluid depletion or bleeding [4, 9].

Hence, all patients at a risk for developing hypovolemia, a narrowing of the LVOT, a small LV lumen, tachycardia, LV hyperkinesia or exposure to catecholamine use may develop IVO.

It is therefore not surprising to see this phenomenon in the ICU setting of sepsis, where an older patient population, frequently bearing a background of chronic hypertension and impaired relaxation, is subject to an acute metabolic and hemodynamic challenge. Septic patients have high circulating catecholamine levels and their intravascular volume is acutely lost to increased permeability, leading to often severe hypovolemia. The treatment instituted in sepsis also adds risk factors: vasopressors used on hypovolemic patients and inotropes both contribute to the hypercontractility, and the frequent use of loop diuretics and sometimes even β-agonists for the treatment of respiratory distress, are all contributing factors to the development of intraventricular obstructive gradients. Moreover, it is an interesting finding of this study that 100 % of the patients with an IVO were already receiving one or more of these (aggravating) therapies.

### Hypovolemia and IVG

The results of this study corroborate the association between hypovolemia and the presence of an intraventricular gradient (LVOT or mid-ventricular) in septic patients, as previously described in the literature in the setting of other hypovolemic states, such as acute blood loss [5, 9].

### Pseudo-LVH

It has been previously demonstrated that, in the setting of decreased filling volumes and reduced end-diastolic diameters, remodeling of LV walls can lead to pseudohypertrophy, in which ventricular walls appear thickened but total ventricular mass remains unchanged when measured by echocardiography or cardiac magnetic resonance imaging [21, 22]. The findings of this study support this as: although there was frequently an appearance of myocardial hypertrophy (thickened LV walls on two-dimensional echocardiography along with the presence of the IVO), in two-thirds of patients the end-diastolic LV area was reduced such that, when myocardial mass was calculated, it actually turned out to be normal - thus 80 % of the time the thickening perceived on two-dimensional echocardiography was pseudo- rather than real hypertrophy.

Therefore, in fluid-depleted patients with decreased end-diastolic diameters, the observation of IVG and thickened walls is more likely to signify hypovolemia rather than true LVH.

### Fluid responsiveness

The fact that the fluid responsiveness observed in this sample of patients with IVG on Doppler flow analysis was close to 90 % constitutes good evidence for performing a fluid challenge on septic shock patients with this echocardiographic finding. Even patients who had no significant increase in cardiac index but who improved their systolic index can still be considered as being in a better overall hemodynamic state since the same cardiac output is now maintained at the expense of less tachycardia. This finding goes along with the previously discussed ones to suggest that the presence of an IVG in a hypotensive septic patient is likely to signify quite severe hypovolemia. Understanding this may be crucial to adequate patient management and to prevent further fluid depletion and catecholamine administration in these patients due to a misinterpretation of their hemodynamic and cardiac status.

### Mortality

Morelli et al. [23] found a high mortality rate in patients with hyperkinesias characterized by a heart rate higher than 95 beats/minute. We may expect that among these patients some may have small and hyperkinetic left ventricles and LV obstruction, as in our study group. The high mortality found in our study may therefore corroborate the high mortality found by Morelli et al. This may be, in part, explained by severe hypovolemia and severity of the sepsis with severe vasodilatation, catecholamines and then hyperkinesia of the left ventricle.

### Study limitations

In this study we did not record echocardiographic imaging in all patients with sepsis, leading to missing data. The protocol in which fluid boluses are given and tracked in our unit does, however, partially help make up for and overcome these impediments, and allowed enough data to be collected for a systematic analysis. A prospective clinical trial, including all patients with septic shock with an echocardiographic recording, would help further establish the parameters for using this method as a formal predictor of fluid responsiveness and may confirm higher mortality of septic shock patients with IVO. The high incidence of IVO may be discussed and may be overestimated because of an “inappropriate” infusion of dobutamine and the fact that cardiac output measurement by the Doppler technique could have been overestimated in cases of IVO.

## Conclusion

This study showed that IVO in the early phase of septic shock is not rare (presenting in over a quarter of septic shock patients) and is associated with a high mortality rate.

Our results warrant caution in the clinical interpretation of LV thickening observed in the setting of hypovolemia and sepsis and support considering the likely possibility of pseudohypertrophy when faced with a small and hypercontractile left ventricle.

The high degree of fluid responsiveness noted in these patients justifies considering a fluid challenge for septic patients with IVO as well as parsimony in the administration of inotropic amines and diuretics.

## Key messages

- At the early phase of septic shock, echocardiographic and Doppler left ventricular obstruction is frequent
- Left ventricular obstruction is associated with hypovolemia in septic shock patients
- Left ventricular obstruction is associated with a high mortality rate.

## Abbreviations

IVG: Intraventricular gradient
IVO: Intraventricular flow obstruction
LV: left ventricular
LVEDD: Left ventricular end-diastolic diameter
LVEF: Left ventricular ejection fraction
LVH: Left ventricular hypertrophy
LVOT: Left ventricular outflow tract
SAM: Systolic anterior movement
SAPS: Simplified Acute Physiology Score
SI: Stroke index
